# Artificial Mineral Waters—Kissingen

**Published:** 1869-11

**Authors:** R. Rother


### ARTIFICIAL MINERAL WATERS—Kissingen.

By R. ROTHER.

The numerous inquiries concerning methods for preparing artificial mineral waters, and the recent attempt of some chemist to furnish the necessary information, together with the growing demand for this luxury, all concur that a commentary upon this interesting subject would not be out of place. As a type of this class of preparations, Kissingen Water was chosen, since the apparent difficulty attending its production, and the high esteem in which it is held, but especially as constituting the topic of an anonymous chemist of the *Druggists' Circular*, make it a point of some interest to the writer.*

That contributor's elaborate and practically impossible process leads to the supposition of being a manufacturer's device, to divert attention from, and further monopolize, the industry of mineral waters; but the indescribable syntax, and worse chemistry, in fact, the total process is so awkwardly exhibited as to betray nothing of the necessary shrewdness usually so characteristic of such an enterprise. The flattering enconium upon the "celebrated Liebig" is cast into ridiculous contrast, when viewed in connection with the author's formula, which, as he states, is to represent Liebig's analysis of the Rakoczy Spring. If it was the design of the author, prompted by a generous impulse, to benefit unassuming enquirers, he should have considered that few can comprehend the abstruse chemical mathematics he lays before them, and that whoever could complete his dubious process would certainly possess the requisite ingenuity to devise a working formula from the original data of the analyst, without soliciting his intervention.

At the present time, the great bulk of artificial mineral waters consumed is furnished by a class of manufacturers who make its fabrication a specialty; each producer claiming for his preparation merits peculiar of its own, representing it as identical with the natural article, but manufactured by entirely original methods, only known to themselves. The retail vender thus obtains his mineral waters in concentrated solution, which, according to manufacturer's direction, is mixed with more water, and the dilute solution impregnated with carbonic acid, under pressure. This is, evidently, a great convenience to those who are not aware of a more economical, but, above all,

* *Druggists' Circular*, July, 1869.

reliable source. What guarantee exists for the correctness of the manufacturer's statement, whose selfishness prompts him to conceal his process? Does the confiding retailer know that the manufacturer's solutions, No. 1 and No. 2, contain *all* the necessary constituents of the mineral water they are to represent? Certainly not, unless he has verified it by analysis, quantitatively as well as qualitatively, which would be an ungrateful task at best, since he has an unquestionable guarantee in the original statement of the analyst who grants it for his love of science alone.

It seems that Liebig's analysis is now usually adopted as the basis for the various methods of preparing Kissingen Water, which, as calculated for the wine pint, or 16 fluid ounces, is stated to contain of solid anhydrous matter and ammonia, as follows:—

| | | |
|---|---|---|
| Chloride Potassium, | 2.2034 | grains. |
| Chloride Sodium, | 44.7133 | " |
| Bromide Sodium, | .644 | " |
| Nitrate Soda, | .0715 | " |
| Chloride Lithium, | .1537 | " |
| Chloride Magnesium, | 2.3331 | " |
| Sulphate Magnesia, | 4.5088 | " |
| Carbonate Magnesia, | .1309 | " |
| Sulphate Lime, | 2.9904 | " |
| Phosphate Lime, | .0431 | " |
| Carbonate Lime, | 8.1482 | " |
| Carbonate Iron, | .2425 | " |
| Silicic Acid, | .0991 | " |
| Ammonia, | .007 | " |

This analysis differs materially from those of some other chemists who have also examined this water, not so much in the total amount of its constituents as in regard to their chemical character and relative quantity; but this is, in some measure, due to the remarkable fluctuations that, from time to time, occur in the composition of some of the mineral springs.

Kastner's analysis is expressed in quantities representing a Bavarian pound, of 16 ounces, or 7680 grains, equal to 560 grammes. The gramme being 15.434 grains Troy, this, by calculation, would then indicate the amount of mineral matter in 8643.04 Troy grains. The slight deviation in the specific gravity of this water above the unit of comparison being practically inappreciable is here left out of consideration. The ratio existing between this bulk (8643.04 grains Troy) and the wine pint (7291.2 grains), when the former is unity, is .84359; this number will then be the coëfficient of the formula when reducing Kastner's result to the wine pint standard.

The presence of an important ingredient, the chloride of lithium, existing even in proportionably large quantity, as found by Liebig and Bauer, was entirely overlooked by Kastner. Liebig also finds a portion of nitrogen in the condition of nitric acid as nitrate of soda, while Kastner found it only as ammonia. The latter chemist also finds much greater quantities of phosphoric acid and protocarbonate of iron, together with alumnia in abundance, but his amount of silicic acid is comparatively enormous. The bromine agrees with, and the chloride of potassium is but half, that of Liebig, yet the remaining and most important components agree within practical limits.

The discrepancies in the analysis of this water evidently occur, then, only among the rarer and least important elements of its composition. The various analysis may, therefore, be considered as identical, when referred to their characteristic bearing upon the main result. This is verefied by the circumstance that Kissingen, made by Kastner's formula, is of the first quality; but since its iron, phosphoric acid, and especially silicic acid, are in excessively large proportion, and the lithia wanting, Liebig's analysis gains the preference. However, this formula, when followed, as indicated by its combination of the elements, yields a result far inferior to that of Kastner.

This is based upon the remarkable fact made apparent by Kastner's distribution of the acids and bases, that sulphate of soda, when not added as such, is not generated in the process, and, consequently, an unpalatable product is the result.

The presence of alumina also exercises a curious influence by concealing the metallic flavor of the iron compound, which, otherwise, is disagreeably perceptible, as with Liebig's formula, where alumina is practically absent.

In offering a working formula for artificial Kissingen Water, the objects in view are simplicity in practice and accuracy in result; a process which, in the hands of even the moderately skilled, shall offer no obstacles to ready execution, and whose constituents shall be within the reach of every pharmaceutist. These are all points not observed by our Brooklyn contemporary, who, very probably, never tested his own process, and, taking his remarks at par, it is very questionable whether he ever tried any other.

Liebig's formula presents only chloride of sodium and nitrate of soda that enter the solution as such; the remainder are brought into it indirectly, resulting from double decompositions between other compounds. This is also true in regard to a portion of the chloride of sodium. Thus, in the working formula, 100 pints are taken as a convenient quantity, that being the capacity of a large fountain. We now assume that the 13.09 grains of protocarbonate of magnesia ($MgO\ CO_2$) (Liebig's number 100 times) reacts with 21.19 grains of sulphate lime ($CaO, SO_3$), forming 15.58 grains of carbonate of lime ($CaO, CO_2$), and 18.7 grains of sulphate of magnesia ($MgO, SO_3$). This decreases the sulphate of lime to 277.85 grains, and increases the carbonate to 830.4 grains, and the sulphate of magnesia to 469.58 grains, while the carbonate of magnesia disappears. This is merely a consolidation to simplify the formula.

For reasons as above stated, Liebig's formula is infringed upon and Kastner's 18 grains of alumina inserted. This determines another complex reaction; 18 grains of alumina are represented by 166.27 of potassa alum. The latter, when decomposed by 78.18 grains of chloride of calcium, and 118.68 grains crystallized carbonate of soda, in succession, yields the alumina, together with 26.08 grains of chloride of potassium, and 61.33 grains of chloride of sodium.

The ammonia is generated as carbonate from 2.2 grains of chloride of ammonium and 5.88 grains of carbonate of soda, 2.41 grains of chloride of sodium also being produced. The employment of bicarbonate is not advisable. It is at best rather indefinite, and not an article of commerce unless prepared from the sesquicarbonate, by exposure to the air. Moreover, it cannot be kept in concentrated solution with the other alkaline salts without incurring loss.

Protocarbonate of iron is obtained from protochloride (first formed by double decomposition between 58.12 grains of protosulphate of iron (crystallized), and 23.25 grains of chloride calcium) and 59.79 grains of carbonate of soda, giving rise to 24.46 grains chloride of sodium.

Phosphate of lime ($3\ CaO, PO_5$), together with 4.88 grains of chloride of sodium is produced from 9.96 grains of crystallized phosphate of soda and 4.63 grains of chloride calcium. If the compound $3\ CaO, PO_5$ is formed, one equivalent of chlorhydric acid must be simultaneously set free. This would instantly react upon the carbonated bases, with the formation of an equivalent of chloride which is not accounted for in surplus of carbonates.

Chloride of lithium is formed when 13.38 grains of carbonate of lithia and 20.07 grains of chloride of calcium react upon each other.

Chloride of lithium is very delinquent, and, hence, indefinite; it is, also, of unfrequent occurrence in commerce, whilst the carbonate is definite, stable, and everywhere obtainable.

Bromide of sodium is supposed to be generated along with 46.65 grains of chloride of potassium, when 74.47 grains of bromide of potassium and 36.58 grains of chloride of sodium are brought in contact, in solution. Bromide of sodium is rarely found in commerce, and the cubical anhydrous never, as these crystals only form at a very low temperature. The bromide of sodium crystallizes, at ordinary temperature, in oblique, rhombic prisms, containing four equivalents of water.

The introduction of the silicic acid offers some difficulty, but the commercial solution of silicate of soda having, usually, a uniform specific gravity is sufficiently accurate, examples examined ranging from 1.384 to 1.388. 104 grains of a solution of the latter specific gravity yielded, after repeated evaporation in contact with chlorhydric acid, 25 grains of silicic acid ($SiO_3$). The residue, by evaporating the filtrate, weighed, after fusion, 21 grains. This 21 grains of chloride of sodium corresponds with 11.128 grains of anhydrous soda (NaO). The solution then contains 36.128 grains of anhydrous silicate of soda, equal to 34.74 per cent.

To determine the formula of this silicate, we have the formula of soda NaO=31, and of silicic acid $SiO_3$=45. The quantity of oxide of sodium divided by its equivalent is 11.128÷31=.359; likewise for silicic acid we have 25÷45=.556. The composition is, therefore, .359 equivalents of NaO to .556 equivalents of $SiO_3$ or .359 : .556 :: 2 (NaO) : 3.1 ($SiO_3$). That is 2 NaO, 3 $SiO_3$. Hence sesquisilicate of soda.

14.46 grains of the anhydrous, or 41.31 grains of the solution of silicate of soda accord with 21 grains of crystalized carbonate of soda.

Sesquibasic silicate of soda (3 NaO, 2 $SiO_3$) is not found in commerce. It is crystalline and indefinite, containing variable quantities of water.

Of the 220.34 grains of chloride of potassium, 72.73 grains have previously been accounted for. The remaining 147.61 grains are supposed to be produced from 172.34 grains of sulphate of potassa and an equivalent quantity of chloride of magnesium.

However abundant chloride of potassium may be in some localities, there is, as yet, no universal demand for it, and, consequently, it is rarely seen. It is also quite deliquescent.

Carbonate of lime is introduced by means of 921.74 grains of chloride of calcium, and 237.94 grains of crystallized carbonate of soda, 971.57 grains of chloride of sodium resulting as a by-product.

Sulphate of lime is furnished by the action of 226.78 grains of chloride of calcium upon 657.85 grains of crystallized sulphate of soda, while 239.03 grains of chloride of sodium are formed.

The exhibition of the magnesian compounds present the most difficult feature, forming, as is apparent, a complexity of the most intricate kind. This, therefore, determines a process by appearance equally comples; but the obstacles are overcome with success, by a subdivision into several phases, independent of each other. 469.58 grains of anhydrous sulphate of magnesia are formed, by assuming that the chloride of magnesium, first obtained by decomposing 962.64 grains of crystallized sulphate of magnesia with 434.36 grains of chloride of calcium, reacts with 172.34 grains of sulphate of potassa and 941.5 grains of crystallized sulphate of soda, giving, as collateral product, 147.61 grains of chloride of potassium and 342.1 grains of chloride of sodium. 233.31 grains of chloride of magnesium results by the mutual decomposition of 604.15 grains of crystallized sulphate of magnesia and 272.61 grains of chloride calcium.

Chloride of magnesium, with 6 equivalents of water, is obtained with difficulty as a very deliquescent crystalline mass, when the solution is evaporated at a moderate heat. Its use is, therefore, practically out of question. The fused anhydrous chloride, were it a commercial article, would, no doubt, answer the purpose, providing its cost was no objection. This is prepared, according to Liebig, by fusing in a platinum crucible the residue left by evaporating a solution containing chloride of ammonium, until the latter is expelled, and the mass in tranquil fusion.

*Fused* chloride of calcium is used in this process, as the crystalline chloride, with 6 equivalents of water, cannot be readily obtained or employed, on account of its extreme tendency to deliquesce.

From the previous preliminary explanations it will be seen that the carbonate of lime, of the lithia reaction, together with the sulphate of lime that results in various double decompositions, with the exception of that generated by a portion of the sulphate of soda, does not enter the mineral water. It is also apparent that the total amount of sulphuric acid is introduced in the condition of sulphates of potassa and soda, 172.34 grains of the former, and 1599.35 grains of the latter. All the magnesium as chloride, obtained from 1566.79 grains of crystallized sulphate of magnesia, all the calcium as chloride, and all the carbonic acid, in combination to protocarbonates, as 2538.29 grains of crystallized carbonate of soda.

The process is now effected by first bringing the entire constituents into simple concentrated solution in two separate parts, designated as "earthy solution," or No. 1, and "alkaline solution, or No. 2. Process No 1 is accomplished in two sections, "*A*" and "*B*," and the course of proceeding takes place as indicated in the following condensed formula:—

EARTHY SOLUTION—(No. 1.)

"*A.*"

| | | |
|---|---|---|
| Chloride of Calcium (fused), - | 1981.62 | grains. |
| Carbonate of Lithia, - - - | 13.38 | " |
| Water, sufficient. | | |

"*B.*"

| | | |
|---|---|---|
| Crystallized Sulphate of Magnesia, - | 1566.79 | grains. |
| " Potassa Alum, - - | 166.27 | " |
| " Protosulphate of Iron, - | 58.12 | " |
| Chloride of Ammonium, - - | 2.2 | " |

ALKALINE SOLUTION—(No. 2.)

| | | |
|---|---|---|
| Chloride of Sodium, - - - | 2862.13 | grains. |
| Crystallized Sulphate of Soda, - | 1599.35 | " |
| " Carbonate of Soda, - | 2538.29 | " |
| Nitrate of Soda, - - - | 7.15 | " |
| Crystallized Phosphate of Soda, - | 9.69 | " |
| Solution of Silicate of Soda, sp. gr. 1.388 (containing 14.46 grains, 2 NaO, 3 $SiO_3$), - - - | 41.31 | " |
| Sulphate of Potassa, - - - - | 172.34 | " |
| Bromide of Potassium, - - - - | 74.47 | " |
| Water, sufficient. | | |

Fractions of a grain above ½ may be considered 1, and less than ½ may be rejected.

Dissolve the chloride of calcium in 12 fluid ounces of water, by trituration, in a mortar, or in a porcelain dish, with heat; to the solution add the carbonate of lithia, and agitate the mixture, then pour the whole into a wide-mouthed bottle, of the capacity of two pints. Dissolve in another vessell, or in the first one, after washing the ingredients of "*B*," namely: sulphate of magnesia, potassa alum, protosulphate of iron, and chloride of ammonium, with one pint of water, and add this to solution "*A*," at once, and in one quantity, the resulting mixture, when the operation is properly managed, will remain clear a few moments, then, gradually, the sulphate of lime separates as a crystalline powder (not amorphous); after five or ten minutes, the whole is thrown on a muslin strainer, and the liquid pressed out with some considerable force; the hard crystalline residue of sulphate of lime, with some carbonate, is then moistened with six or eight fluid ounces of water, and again wrung out. The nearly clear solution thus obtained will measure about two pints, and may be filtered, if desirable, which, however, is altogether superfluous. This constitutes solution No. 1.

The chloride of sodium (pure and perfectly dry), is now dissolved in 1½ pints of water, with frequent agitation. The sulphates of potassa and soda, and the carbonate of soda (dry, but not effloresced), are together dissolved in somewhat less than 1½ pints of water, with the aid of heat; to this is added the chloride of sodium solution, and then the remaining salts, first dissolved in a few fluid ounces of water- This furnishes solution No. 2, measuring about 3 pints, which must be filtered.

Solutions No. 1 and No. 2, when added in succession to 12 gallons of water in a fountain, and impregnated with carbonic acid, under a desirable pressure, yield artificial Kissingen Water of unsurpassed quality. These results are based upon the writer's own experience, who has, during the past season, frequently manufactured this mineral water by the above process, in quantities of ten fountains at a time.

The writer, therefore, considers his duty done; let subsequent operators now do theirs.—*The Chicago Pharmacist.*

